# The effect of surgery on primary splenic lymphoma: A study based on SEER database

**DOI:** 10.1002/cam4.4238

**Published:** 2021-09-21

**Authors:** Xiaotao Pan, Dongfeng Ren, Ya Li, Jin Zhao

**Affiliations:** ^1^ Department of General Surgery Shaanxi Provincial Cancer Hospital Xi'an China; ^2^ Department of Oncology The First Hospital of Yulin Yulin China; ^3^ Department of Oncology Shaanxi Provincial Cancer Hospital Xi'an China; ^4^ Department of Radiotherapy Shaanxi Provincial Cancer Hospital Xi'an China

**Keywords:** primary splenic lymphoma, propensity score matching, SEER, surgery

## Abstract

**Background:**

Although primary splenic lymphoma (PSL) is rare, it ranks first among splenic primary malignant cancers, and the incidence of lymphoma of spleen has gradually increased in recent years. However, the efficacy of surgery for PSL has not been clinically verified by large sample data, which has affected the formulation of relevant guidelines.

**Aim:**

To assess whether surgery can enhance the prognosis PSL patients.

**Methods:**

Extracted the data of patients with PSL from The Surveillance, Epidemiology, and End Results (SEER) database, and divided the patients into surgery and non‐surgery group. Kaplan–Meier curves and log‐rank tests were used to compare the overall survival (OS) and cancer‐specific survival (CSS). The propensity score matching (PSM) was used to match the data, then compared the OS and CSS again. The COX proportional hazard regression model was used for univariate and multivariate analysis. Finally, we performed subgroup analysis in different Ahmann stages.

**Results:**

A sum of 2207 patients with PSL were enrolled, of which 1062 (48.1%) patients received surgery, and 1145 (51.9%) patients did not undergo surgery. Overall, patients in the surgery group had better OS and CSS. After the propensity scores matching, surgery was not statistically significant in OS and CSS. In the subgroup analysis, surgery was a protective factor for the OS and CSS in Ahmann I/II. However, surgery was no statistical significance in OS and CSS in Ahmann III. In patients with Ahmann Ⅰ/Ⅱ SMZL, surgery was a protective factor for OS and CSS. In patients with Ahmann Ⅲ SMZL, surgery was also statistically significant of OS and CSS.

**Conclusions:**

Surgery can significantly improve the prognosis of patients with Ahmann Ⅰ/Ⅱ primary splenic lymphoma, but there was no survival difference in the Ahmann Ⅲ patients with or without surgery. For patients with SMZL, surgery was effective for improving OS and CSS.

## Graphical abstract

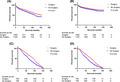

## 1 INTRODUCTION

PSL is rare and it accounts for only about 1% of all malignant tumors, but it ranks first among splenic primary malignant cancers, and the incidence of lymphoma of spleen has gradually increased in recent years.^1^, ^2^, ^3^, ^4^ The current studies on PSL are mainly case reports. These studies show that the most common symptoms of primary splenic lymphoma are malaise, weight loss, and fever, with non‐Hodgkin's lymphoma predominantly.^5^, ^6^, ^7^, ^8^, ^9^ Infection after Hepatitis C is more likely to develop primary splenic lymphoma. This may be related to the uncontrolled proliferation of lymphocytes after the hepatitis virus infects the spleen due to the body's susceptibility and genetic mutations.^10^, ^11^, ^12^, ^13^, ^14^, ^15^, ^16^

The diagnostic criteria for primary splenic lymphoma have gone through a process from strict to simple. Currently, the most commonly used is Ahmann stage,^17^ Ahmann I tumors are completely confined to the spleen and do not involve hilar lymph nodes and distant organs; Ahmann II tumors involve hilar lymph nodes; Ahmann III tumors have distant metastases such as liver, abdominal lymph nodes, and even bone marrow. Therefore, according to this stage, the corresponding treatment method is derived: Ahmann I is feasible with simple splenectomy; Ahmann II splenectomy plus splenic hilar lymph node dissection, postoperative chemotherapy, or local radiotherapy; Ahmann III comprehensive treatment based on surgery.

At present, there is no unified diagnosis and treatment guidelines for primary splenic lymphoma. This study intends to verify the efficacy of surgery for primary splenic lymphoma through large sample data.

## 2 METHODS

### 2.1 Data sources

Extracted the general data, pathological type, TNM stage, treatment data (surgery, radiotherapy, and chemotherapy), and survival data of all primary splenic lymphoma individuals in the SEER database.

### 2.2 Inclusion criteria

Patients older than 18 years; Diagnosis of primary splenic lymphoma based on pathological diagnosis.

### 2.3 Exclusion criteria

Patients data obtained from autopsies and only death reports; Cases with incomplete follow‐up, survival time, and survival outcome.

### 2.4 Stage conversion

Since there is only the AJCC stage of lymphoma in the SEER database, the AJCC stage need to be transformed into Ahmann stage according to their stage standards. AJCC I corresponds to Ahmann I or II, AJCC II, III, and IV correspond to Ahmann Phase III.

### 2.5 Statistical analysis

The *t*‐test was used for measurement data; the Chi‐square test was used for classification data; and the Mann–Whitney *U* test was used when the conditions for using measurement and classification data were not met in the baseline data comparison. Then PSM was used. The Kaplan–Meier curves and log‐rank test were used to compare the OS and CSS difference before and after PSM. OS was the period from the diagnosis of primary splenic lymphoma to death of any causes; CSS was the period from diagnosis of primary splenic lymphoma to death from primary splenic lymphoma. The Cox proportional hazards regression model was also used to explore whether surgery is a risk factor for the OS and CSS of PSL patients. Finally, we performed subgroup analysis in different Ahmann stages.

SPSS23.0 (IBM Corp.,) was used in the process of statistical analysis, for statistically significant *p* values, choose 0.05 on both sides.

## 3 RESULTS

### 3.1 Comparison of baseline data

A sum of 2207 patients with primary splenic lymphoma were included, including 1062 patients in the surgical group and 1145 patients in the non‐surgical group. In the surgical group, the proportion of patients aged ≤65 years was higher (56.1% vs. 43.2%, *p *< 0.001), the proportion of marriage was higher (64.5% vs. 60.3%, *p *= 0.044), and the proportion of Ahmann Ⅰ/Ⅱ was higher (40.9% vs. 19.4%, *p *< 0.001), the proportion of receiving radiotherapy was lower (2.3% vs. 3.8%, *p *= 0.032), and the proportion of receiving chemotherapy was lower (42.9% vs. 57.6%, *p *< 0.001). There was no statistical difference between the two groups in the distribution of sex (*p *= 0.056), race (*p *= 0.791), and pathological type (*p *= 0.371). (Table 1).

**TABLE 1:** Comparison of baseline data

Characteristics	Total	Surgery	No Surgery	*p*
	2207	*N* = 1062 (48.1%)	*N* = 1145 (51.9%)	
Age				
≤65	1091	596 (56.1%)	495 (43.2%)	**<0.001**
>65	1116	466 (43.9%)	650 (56.8%)	
Sex				
Male	1145	517 (48.7%)	604 (52.8%)	0.056
Female	1062	545 (51.3%)	541 (47.2%)	
Race				
White	1957	942 (88.7%)	1015 (88.6%)	0.791
Black	141	65 (6.1%)	76 (6.6%)	
Other	109	55 (5.2%)	54 (4.7%)	
Marital status				
No married	831	377 (35.5%)	454 (39.7%)	**0.044**
Married	1376	685 (64.5%)	691 (60.3%)	
Ahmann stage				
Ⅰ/Ⅱ	656	434 (40.9%)	222 (19.4%)	**<0.001**
Ⅲ	1551	628 (59.1%)	923 (80.6%)	
Pathological type				
B	2102	1007 (94.8%)	1095 (95.6%)	0.371
T	105	55 (5.2%)	50 (4.4%)	
Radiation				
No	2139	1038 (97.7%)	1101 (96.2%)	**0.032**
Yes	68	24 (2.3%)	44 (3.8%)	
Chemotherapy				
No	1092	606 (57.1%)	486 (42.4%)	**<0.001**
Yes	1115	456 (42.9%)	659 (57.6%)	

Bold indicate *p * values < 0.05 are statistically significant.

### 3.2 Propensity score matching

Incorporate above variables data to 1:1 PSM. After PSM, there were 785 patients in each group and the characteristics were no longer significantly different: age (*p* = 0.686), sex (*p* = 0.960), race (*p* = 0.623), marital status (*p* = 0.677), Ahmann stage (*p* = 0.605), Pathological type (*p* = 1.000), radiotherapy (*p* = 1.000), and chemotherapy (*p* = 0.614). (Table 2).

**TABLE 2:** Comparison of baseline data after PSM

Characteristics	Total	Surgery	No Surgery	P
	1570	*N* = 785 (50.0%)	*N* = 785 (50.0%)	
Age				
≤65	806	407 (51.8%)	399 (50.8%)	0.686
>65	764	378 (48.2%)	386 (49.2%)	
Sex				
Male	783	392 (49.9%)	391 (49.8%)	0.960
Female	787	393 (50.1%)	394 (50.2%)	
Race				
White	1387	695 (88.5%)	692 (88.2%)	0.623
Black	100	46 (5.9%)	54 (6.9%)	
Other	83	44 (5.6%)	39 (5.0%)	
Marital status				
No married	594	301 (38.3%)	293 (37.3%)	0.677
Married	976	484 (61.7%)	492 (62.7%)	
Ahmann stage				
Ⅰ/Ⅱ	409	200 (25.5%)	209 (26.6%)	0.605
Ⅲ	1161	585 (74.5%)	576 (73.4%)	
Pathological type				
B	1512	756 (96.3%)	756 (96.3%)	1.000
T	58	29 (3.7%)	29 (3.7%)	
Radiation				
No	1538	769 (98.0%)	769 (98.0%)	1.000
Yes	32	16 (2.0%)	16 (2.0%)	
Chemotherapy				
No	804	407 (51.8%)	397 (50.6%)	0.614
Yes	766	378 (48.2%)	398 (49.4%)	

### 3.3 Comparison of OS and CSS

**FIGURE 1:**
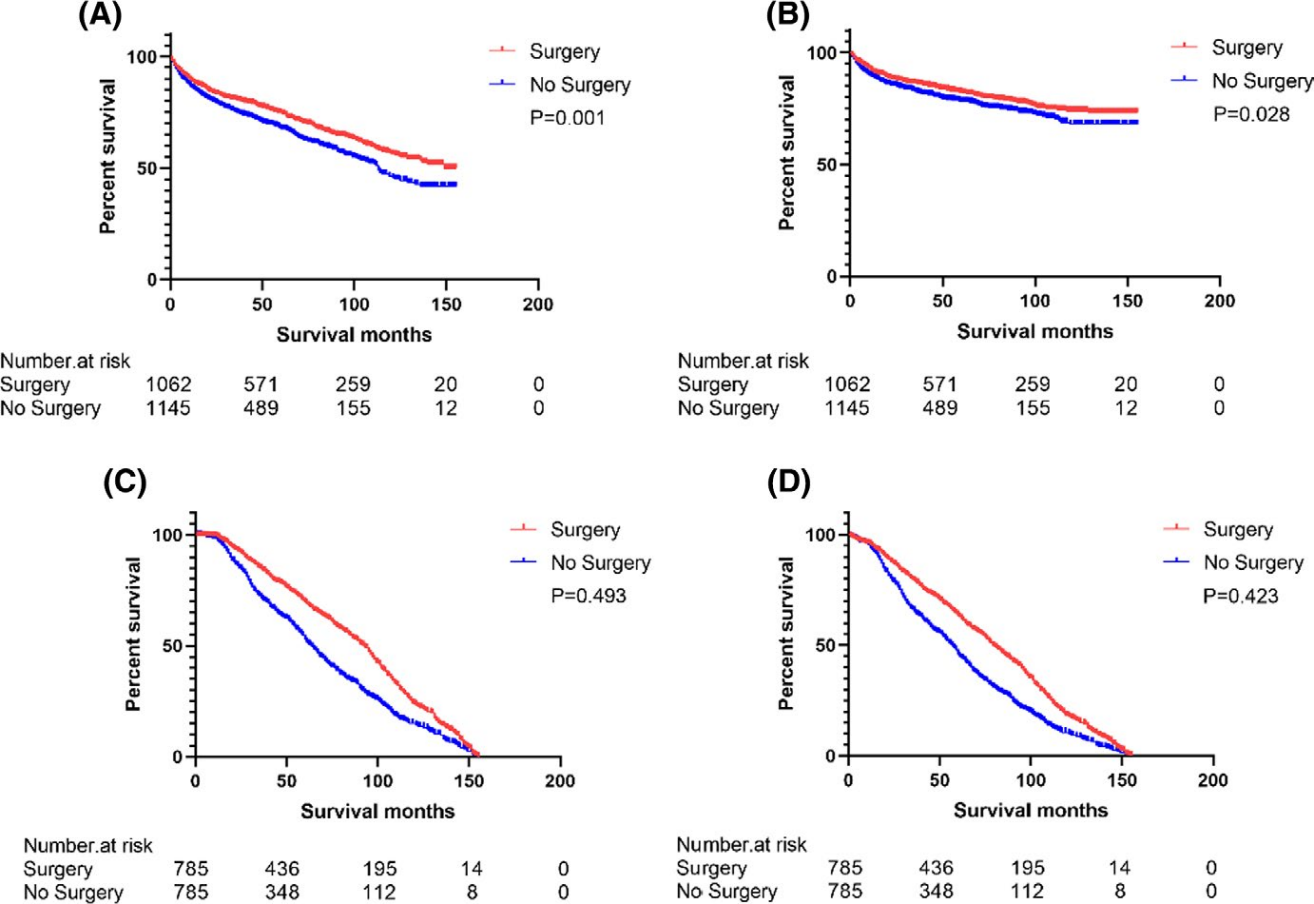
Survival curve for OS and CSS comparison. OS (A) and CSS (B) for primary splenic lymphoma patients before PSM; OS (C) and CSS (D) for primary splenic lymphoma patients after PSM

Table 3 showed comparison of OS and CSS of primary splenic lymphoma patients. Before PSM, among the patients with primary splenic lymphoma, the surgical group had better OS (HR = 0.783, 95% CI: 0.680–0.901, *p* = 0.001) (Figure 1A) and CSS (HR = 0.817, 95% CI: 0.683–0.978, *p* = 0.028) (Figure 1B) than the non‐surgical group; After PSM, in the overall patients with primary splenic lymphoma, the OS (HR = 0.943, 95% CI: 0.798–1.114, *p* = 0.493) (Figure 1C) and CSS (HR = 1.091, 95% CI: 0.881–1.351, *p* = 0.423) (Figure 1D) of the two groups of patients were not statistically different;

**TABLE 3:** Comparison of overall survival and cancer‐specific survival

	Characteristics	Overall survival		Cancer‐specific survival	
		HR (95% CI)	*p*	HR (95% CI)	*p*
Before PSM	No Surgery	Reference		Reference	
	Surgery	0.783 (0.680, 0.901)	**0.001**	0.817 (0.683,0.978)	**0.028**
After PSM	No Surgery	Reference		Reference	
	Surgery	0.943 (0.798, 1.114)	0.493	1.091 (0.881,1.351)	0.423

Bold indicate *p * values < 0.05 are statistically significant.

Abbreviation: CI, Confident interval; HR, Hazard ratio.

### 3.4 Univariate and multivariate analysis of OS and CSS

Table 4 showed that the results of survival analysis of the OS of patients with PSL. In univariate analysis, age>65 (HR = 2.463, 95% CI: 2.213–2.858, *p *< 0.001), Ahmann Ⅲ (HR = 1.216, 95% CI: 1.037–1.425, *p* = 0.016), and T‐cell lymphoma (HR = 3.644, 95% CI: 2.863–4.639, *p *< 0.001) were the risk factors for the OS of patients with PSL; Marriage (HR = 0.712, 95% CI: 0.619–0.819, *p* < 0.001) and surgery (HR = 0.783, 95% CI: 0.680–0.901, *p* = 0.001) were protective factors for the OS of patients with PSL; However, sex, race, radiotherapy, and chemotherapy were not statistically significant. (*p* values are greater than 0.05).

**TABLE 4:** Univariate and multivariate analysis of overall survival

Characteristics	Univariate analysis	*p*	Multivariate analysis	*p*
	HR (95% CI)		HR (95% CI)	
Age				
≤65	Reference		Reference	
>65	2.463 (2.213, 2.858)	**<0.001**	2.939 (2.510, 3.441)	**<0.001**
Sex				
Male	Reference			
Female	0.998 (0.869, 1.147)	0.982		
Race				
White	Reference			
Black	1.148 (0.872, 1.511)	0.325		
Other	1.053 (0.766, 1.488)	0.750		
Marital status				
No married	Reference		Reference	
Married	0.712 (0.619, 0.819)	**<0.001**	0.722 (0.627, 0.832)	**<0.001**
Ahmann stage				
Ⅰ/Ⅱ	Reference		Reference	
Ⅲ	1.216 (1.037, 1.425)	**0.016**	1.207 (1.024, 1.423)	**0.025**
Pathological type				
B	Reference		Reference	
T	3.644 (2.863, 4.639)	**<0.001**	5.778 (4.474, 7.463)	
Surgery				
No	Reference		Reference	
Yes	0.783 (0.680, 0.901)	**0.001**	0.905 (0.781, 1.047)	0.180
Radiation				
No	Reference			
Yes	1.210 (0.824, 1.776)	0.331		
Chemotherapy				
No	Reference			
Yes	1.085 (0.944, 1.248)	0.249		

Bold indicate *p * values < 0.05 are statistically significant.

Abbreviations: CI, Confident interval; HR, Hazard ratio.

**FIGURE 2:**
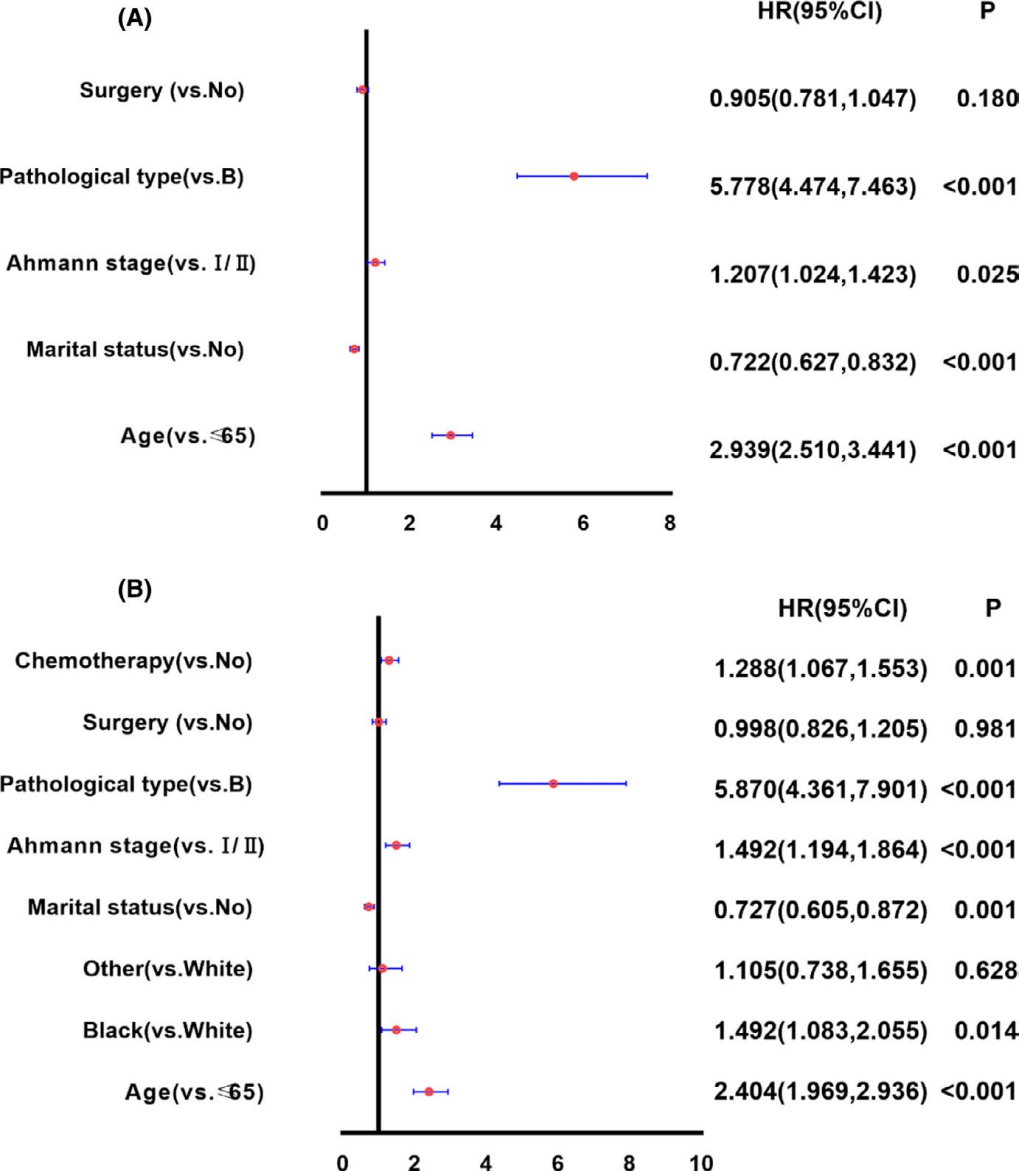
Forestmap of multivariate analysis of OS and CSS. (A) Multivariate analysis of overall survival of primary splenic lymphoma; (B) Multivariate analysis of cancer‐specific survival of primary splenic lymphoma

Incorporated statistically significant variables in the univariate analysis into the regression model for multivariate analysis, the results showed that age > 65 (HR = 2.939, 95% CI: 2.510–3.441, *p* < 0.001), Ahmann Ⅲ (HR = 1.207, 95% CI: 1.024–1.423, *p* = 0.025), and T‐cell lymphoma (HR = 5.778, 95% CI: 4.474–7.463, *p* < 0.001) were risk factors for the OS of patients with PSL; Marriage (HR = 0.722, 95% CI: 0.627–0.832, *p* < 0.001) was a protective factor for the OS of patients with PSL; Surgery (HR = 0.905, 95% CI: 0.781–1.047, *p* = 0.180) was not statistically significant. (Figure 2A).

Table 5 showed that the results of survival analysis of the CSS of patients with PSL. In univariate analysis, age > 65 (HR = 1.791, 95% CI: 1.491–2.152, *p* < 0.001), black (HR = 1.644, 95% CI: 1.212–2.230, *p* = 0.001), Ahmann Ⅲ (HR = 1.513, 95% CI: 1.220–1.877, *p* < 0.001), T‐cell lymphoma (HR = 4.774, 95% CI: 3.648–6.248, *p* < 0.001), and chemotherapy (HR = 1.366, 95% CI: 1.140–1.636, *p* = 0.001) were the risk factors for the CSS of patients with primary splenic lymphoma; Marriage (HR = 0.677, 95% CI: 0.566–0.809, *p* < 0.001) and surgery (HR = 0.817, 95% CI: 0.683–0.978, *p* = 0.028) were protective factors for the CSS of patients with primary splenic lymphoma; However, sex and radiotherapy were not statistically significant. (*p* values are greater than 0.05).

**TABLE 5:** Univariate and multivariate analysis of cancer‐specific survival

Characteristics	Univariate analysis	*p*	Multivariate analysis	*p*
	HR (95% CI)		HR (95% CI)	
Age				
≤65	Reference		Reference	
>65	1.791 (1.491, 2.152)	**<0.001**	2.404 (1.969,2.936)	**<0.001**
Sex				
Male	Reference			
Female	0.926 (0.774, 1.106)	0.396		
Race				
White	Reference		Reference	
Black	1.644 (1.212, 2.230)	**0.001**	1.492 (1.083,2.055)	**0.014**
Other	1.165 (0.784, 1.732)	0.449	1.105 (0.738,1.655)	0.628
Marital status				
No married	Reference		Reference	
Married	0.677 (0.566, 0.809)	**<0.001**	0.727 (0.605,0.872)	**0.001**
Ahmann stage				
Ⅰ/Ⅱ	Reference		Reference	
Ⅲ	1.513 (1.220, 1.877)	**<0.001**	1.492 (1.194,1.864)	**<0.001**
Pathological type				
B	Reference		Reference	
T	4.774 (3.648, 6.248)	**<0.001**	5.870 (4.361,7.901)	**<0.001**
Surgery				
No	Reference		Reference	
Yes	0.817 (0.683, 0.978)	**0.028**	0.998 (0.826,1.205)	0.981
Radiation				
No	Reference			
Yes	1.444 (0.923, 2.260)	0.107		
Chemotherapy				
No	Reference		Reference	
Yes	1.366 (1.140, 1.636)	**0.001**	1.288 (1.067,1.553)	**0.001**

Bold indicate *p * values < 0.05 are statistically significant.

Abbreviations: CI, Confident interval; HR, Hazard ratio.

Multivariate analysis in the same way and the results showed that age > 65 (HR = 2.404, 95% CI: 1.969–2.936, *p* < 0.001), black (HR = 1.492, 95% CI: 1.083–2.055, *p* = 0.014), Ahmann Ⅲ (HR = 1.492, 95% CI: 1.194–1.864, *p* < 0.001), T‐cell lymphoma (HR = 5.870, 95% CI: 4.361–7.901, *p* < 0.001), and chemotherapy (HR = 1.288, 95% CI: 1.067–1.553, *p* = 0.001) were risk factors for the CSS of patients with primary splenic lymphoma; Marriage (HR = 0.727, 95% CI: 0.605–0.872, *p* = 0.001) was a protective factor for the CSS of patients with primary splenic lymphoma; Surgery (HR = 0.998, 95% CI: 0.826–1.205, *p* = 0.981) was not statistically significant. (Figure 2B).

### 3.5 Subgroup analysis stratified by Ahmann stage

**FIGURE 3:**
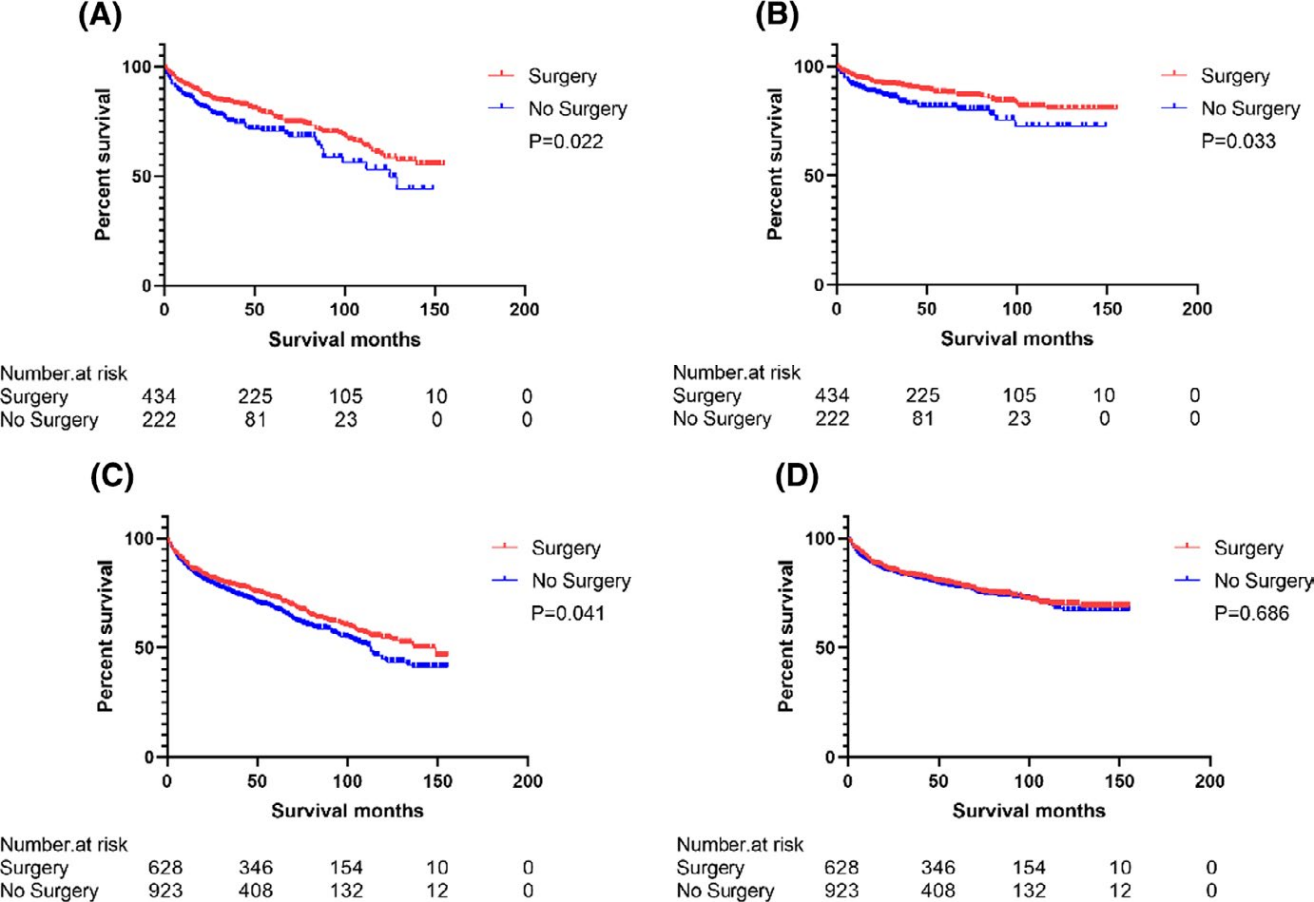
Survival curve for subgroup OS and CSS comparison. OS (A) and CSS (B) for Ahmann Ⅰ/Ⅱ primary splenic lymphoma patients. OS (C) and CSS (D) for Ahmann Ⅲ primary splenic lymphoma patients

Table 6 showed that primary splenic lymphoma survival analysis of OS and CSS stratified by Ahmann stage. In univariate analysis, among patients with Ahmann Ⅰ/Ⅱ primary splenic lymphoma, the surgical group had better OS (HR = 0.716, 95% CI: 0.528–0.952, *p* = 0.022) (Figure 3A) and CSS (HR = 0.654, 95% CI: 0.442–0.967, *p* = 0.033) (Figure 3B) than the non‐surgical group; Among patients with Ahmann Ⅲ primary splenic lymphoma, the surgical group had better OS (HR = 0.841, 95% CI: 0.713–0.993, *p* = 0.041) (Figure 3C) than the non‐surgical group, but there was no statistical difference in CSS (HR = 0.959, 95% CI: 0.781–1.177, *p* = 0.686) (Figure 3D); In the multivariate analysis, the results showed that in patients with Ahmann Ⅰ/Ⅱ primary splenic lymphoma, surgery was a protective factor for OS (HR = 0.740, 95% CI: 0.551–0.994, *p* = 0.046) and CSS (HR = 0.658, 95% CI: 0.442–0.980, *p* = 0.039). In patients with Ahmann Ⅲ primary splenic lymphoma, surgery was not statistically significant in the multivariate analysis of OS (HR = 0.973, 95% CI: 0.822–1.153, *p* = 0.755) and CSS (HR = 1.101, 95% CI: 0.894–1.357, *p* = 0.366).

**TABLE 6:** Subgroup univariate and multivariate analysis of OS and CSS

	Ahmann stage	Characteristics	Univariate analysis		Multivariate analysis	
			HR (95% CI)	*p*	HR (95% CI)	*p*
OS	Ⅰ/Ⅱ	No Surgery	Reference		Reference	
		Surgery	0.716 (0.528,0.952)	**0.022**	0.740 (0.551,0.994)	**0.046**
	Ⅲ	No Surgery	Reference		Reference	
		Surgery	0.841 (0.713,0.993)	**0.041**	0.973 (0.822,1.153)	0.755
CSS	Ⅰ/Ⅱ	No Surgery	Reference		Reference	
		Surgery	0.654 (0.442,0.967)	**0.033**	0.658 (0.442,0.980)	**0.039**
	Ⅲ	No Surgery	Reference		Reference	
		Surgery	0.959 (0.781,1.177)	0.686	1.101 (0.894,1.357)	0.366

Bold indicate *p * values < 0.05 are statistically significant.

Abbreviations: CI, Confident interval; HR, Hazard ratio.

### 3.6 Survival analysis of patients with splenic marginal zone lymphoma (SMZL) stratified by Ahmann stage

Table 7 showed survival analysis of patients with SMZL stratified by Ahmann stage. In this study, there were a total of 1140 SMZL patients, so a subgroup analysis was performed on this type of patients, while the number of samples from patients with other pathological types of primary splenic lymphoma was small, so subgroup analysis was not performed. In univariate analysis, among patients with Ahmann Ⅰ/Ⅱ SMZL, the surgical group had better OS (HR = 0.492, 95% CI:0.356–0.680, *p* < 0.001) and CSS (HR = 0.554, 95% CI:0.418–0.736, *p* < 0.001) than the non‐surgical group; Among patients with Ahmann Ⅲ SMZL, the surgical group had better OS (HR = 0.730, 95% CI:0.618–0.862, *p* < 0.001) than the non‐surgical group and CSS (HR = 0.686, 95% CI:0.589–0.799, *p* < 0.001); In the multivariate analysis, the results showed that in patients with Ahmann Ⅰ/Ⅱ SMZL, surgery was a protective factor for OS (HR = 0.463, 95% CI:0.325–0.660, *p* < 0.001) and CSS (HR = 0.564, 95% CI:0.415–0.765, *p* < 0.001). In patients with Ahmann Ⅲ SMZL, surgery was also statistically significant in the multivariate analysis of OS (HR = 0.690, 95% CI:0.579–0.823, *p* < 0.001) and CSS (HR = 0.676, 95% CI:0.575–0.794, *p* < 0.001).

**TABLE 7:** Subgroup analysis of OS and CSS in SMZL patients

	Ahmann stage	Characteristics	Univariate analysis		Multivariate analysis	
			HR (95% CI)	*p*	HR (95% CI)	*p*
OS	Ⅰ/Ⅱ	No Surgery	Reference		Reference	
		Surgery	0.492 (0.356,0.680)	**<0.001**	0.463 (0.325,0.660)	**<0.001**
	Ⅲ	No Surgery	Reference		Reference	
		Surgery	0.730 (0.618,0.862)	**<0.001**	0.690 (0.579,0.823)	**<0.001**
CSS	Ⅰ/Ⅱ	No Surgery	Reference		Reference	
		Surgery	0.554 (0.418,0.736)	**<0.001**	0.564 (0.415,0.765)	**<0.001**
	Ⅲ	No Surgery	Reference		Reference	
		Surgery	0.686 (0.589,0.799)	**<0.001**	0.676 (0.575,0.794)	**<0.001**

Bold indicate *p * values < 0.05 are statistically significant.

Abbreviations: CI, Confident intervalHR, Hazard ratio.

## 4 DISCUSSION

The diagnostic criteria for primary splenic lymphoma have gone through a process from strict to simple. In 1965, Das‐Gupta et al. first proposed the diagnostic criteria. Due to the emphasis on symptoms, the early symptoms of primary splenic lymphoma may not be typical, so the diagnosis rate was not high^18^; Later, Kehoe et al. proposed that primary splenic lymphoma can be accompanied by involvement of splenic hilar lymph nodes, liver, or bone marrow. It was mainly based on the involvement of the site to infer whether the spleen was the primary site. This diagnostic standard made the diagnosis rate of primary splenic lymphoma greatly increased^19^; Currently, the most commonly used is Ahmann stage.^17^ In Ahmann stage, different treatment methods are recommended according to different stages of primary splenic lymphoma, but there is a lack of large sample data for verification. Therefore, this study is based on a database to verify the efficacy of surgery for primary splenic lymphoma.

Previous small sample studies have shown that patients with PSL have a good prognosis after splenectomy.^20^, ^21^, ^22^ The research of Sachin et al. showed splenectomy prevented potentially serious complications related to hypersplenism and splenic rupture.^23^ Research by Kraus et al. also showed that splenectomy can not only be used for treatment, but also an important diagnostic method for primary splenic lymphoma.^24^ According to previous animal experiments, splenectomy was an effective way to treat canine splenic lymphoma.^25^ Julien Lenglet et al. conducted a long‐term follow‐up of 100 SMZL patients undergoing splenectomy. The results showed that splenectomy could effectively correct the cytopenia and lymphocytosis, so there was a better OS; At the same time, the primary tumor lesions were removed and the risk of tumor metastasis was reduced, so there was better progression free survival (PFS).^26^ A small sample study from Norway also showed that after splenectomy, the local and cytopenia symptoms of PSL patients improved.^22^ In the patient population of Ahmann Ⅰ/Ⅱ primary splenic lymphoma, the results of univariate and multivariate analysis showed that surgery were a protective factor for OS and CSS. In the patient population of Ahmann Ⅲ primary splenic lymphoma, the results of univariate and multivariate analysis showed that surgery were not statistically significant. These results collectively indicate that surgery can improve the prognosis of patients with primary splenic lymphoma of Ahmann Ⅰ/Ⅱ, but was of little significance for the prognosis of patients with Ahmann Ⅲ. In this study, for the overall patients with primary splenic lymphoma, the results of survival analysis after PSM and multivariate analysis showed that surgery was not statistically significant. The reason may be that in the cases included in this study, Ahmann Ⅲ patients accounted for much more than Ahmann Ⅰ/Ⅱ patients, thus reducing the overall efficacy of surgery. This was consistent with previous research results: the prognosis of patients with stage Ⅲ primary splenic lymphoma after splenectomy was worse than that of stages Ⅰ and Ⅱ, but there were 15 cases in this study.^19^ However, for SMZL patients, splenectomy can not only improve the prognosis of stage Ⅰ and Ⅱ, but also a protective factor for the survival of stage Ⅲ. Therefore, surgical treatment should be actively performed for SMZL patients who meet the conditions for surgical treatment.

In the past few decades, splenectomy was the first‐line treatment for SMZL. In the past decade, an in‐depth understanding of the pathobiology and molecular characteristics of SMZL has led to the development of many new treatment options, for example, rituximab monotherapy with or without splenectomy, and rituximab with chemotherapy and splenectomy alone are the current first‐line treatment options for SMZL. However, the current understanding of the molecular characteristics of SMZL is still limited, which is the direction and difficulty of future research.^27^, ^28^

The results of this study also showed that T‐cell lymphoma had a worse prognosis. This was consistent with the results of previous animal experiments.^25^ The results of other previous studies also showed that: Splenic B‐cell lymphoma had a better prognosis. This may be that T‐cell‐derived lymphomas were often aggressive and prone to distant metastasis. They were often accompanied by bone marrow infiltration at the time of diagnosis, and their 5‐year survival rate was very low, so the prognosis was very poor.^28^, ^29^, ^30^, ^31^

This study is based on large sample data to verify the efficacy of surgery for primary splenic lymphoma, fills the gap in the clinical research, and can provide a reference for the formulation of clinical guidelines for primary splenic lymphoma. This study has some limitations. This study was a retrospective cohort study and the database data, for example, laboratory and imaging examination data, were not complete, and it was also impossible to get information on whether the tumor recurred and complications. Therefore, prospective cohort studies and basic research are needed to verify the results of this study.

## 5 CONCLUSIONS

Surgery can significantly improve the prognosis of patients with Ahmann Ⅰ/Ⅱ primary splenic lymphoma, but there was no survival difference in the Ahmann Ⅲ patients with or without surgery. For patients with SMZL, surgery was effective for improving OS and CSS.

## CONFLICTS OF INTEREST

The authors declare no conflicts of interest.

## ETHICS APPROVAL AND CONSENT TO PARTICIPATE

This retrospective study was approved by the Ethics Committee of Shaanxi Provincial Cancer Hospital. The study was based on database data and the patient's specific identity cannot be known, so the patient's informed consent was not required.

## Data Availability

The data of this study are available by requesting from the corresponding author.
